# Antimicrobial Use and Susceptibility of Indicator *Escherichia coli* in Finnish Integrated Pork Production

**DOI:** 10.3389/fmicb.2021.754894

**Published:** 2021-11-04

**Authors:** Virpi Sali, Suvi Nykäsenoja, Annamari Heikinheimo, Outi Hälli, Taneli Tirkkonen, Mari Heinonen

**Affiliations:** ^1^Department of Production Animal Medicine, University of Helsinki, Mäntsälä, Finland; ^2^Microbiology Unit, Finnish Food Authority, Helsinki, Finland; ^3^Department of Food Hygiene and Environmental Health, University of Helsinki, Helsinki, Finland; ^4^Atria Finland Plc, Seinäjoki, Finland

**Keywords:** antimicrobial use, antimicrobial resistance, pig herd, indicator organism, integrated pork production

## Abstract

In pigs, antimicrobial use (AMU) practices vary at different production phases between herds and between countries. Antimicrobial resistance (AMR) development is linked to AMU but recognized as a multi-factorial issue, and thus, any information increasing knowledge of AMU and AMR relationships is valuable. We described AMU and screened the carriage of different AMR phenotypes of indicator *Escherichia coli* in 25 selected Finnish piglet-producing and finishing herds that formed nine birth-to-slaughter production lines. Moreover, we studied associations between AMU and AMR in both herd types and throughout the production line. Treatment records were obtained from the national Sikava register for 1year, and AMU was quantified as mg/PCU (population correction unit) and TIs (treatment incidences). For phenotypic antimicrobial susceptibility testing, ten pen-level pooled feces samples (*n*=250) in each herd were collected from one room representing the oldest weaned piglets or the oldest finishing pigs. Majority of the medications (96.8%) was administered parenterally, and penicillin was the predominant antimicrobial in every herd. More different antimicrobial substances were used in piglet-producing than in finishing herds (median 5 and 1, respectively, *p*<0.001). As mg/PCU, sows had the highest AMU and suckling piglets had the highest TIs, whereas finishing pigs were the least treated age group. The proportion of susceptible indicator *E. coli* isolates of all studied isolates was 59.6%. Resistance was found most commonly against tetracycline, sulfamethoxazole, trimethoprim, and ampicillin, and multi-resistant (MR) isolates (46.5% of all resistant isolates) were resistant to a maximum of four different antimicrobials. Quinolone resistance was rare, and no resistance against 3rd-generation cephalosporins, meropenem, azithromycin, colistin, gentamicin, or tigecycline was detected. The main associations between AMU and AMR were found at antimicrobial group level when use was compared with the presence of AMR phenotypes. The proportion of resistant isolates was not associated with AMU, and herd size was not associated with either AMU or AMR. We suggest that the use of narrow-spectrum beta-lactams as a primary treatment option and lack of wide application of oral group medications potentially favors a good resistance pattern in integrated pork production.

## Introduction

In Finland, the prevalence of infectious pig diseases is generally low (Finnish Food Authority, 2018), and veterinary antimicrobial use (AMU) has been one of the lowest in the European Union for years (ESVAC, 2020). Similar to other European countries, pigs are in general the major production animal species treated with antimicrobials in Finland (ESVAC, 2020). Nevertheless, AMU practices have been reported to differ between countries (Chantziaras et al., 2014; De Briyne et al., 2014; Garcia-Migura et al., 2014; Sjölund et al., 2016; Sarrazin et al., 2019; ESVAC, 2020; O’Neill et al., 2020, herds (Moreno, 2014), and production systems (Moreno, 2012; Makita et al., 2016). Antimicrobial resistance (AMR) is a globally growing problem (WHO, 2019a), and all AMU sets selection pressure for AMR (Asai et al., 2005; Harada et al., 2008; Faldynova et al., 2013; Chantziaras et al., 2014; JIACRA, 2015; Makita et al., 2016; Callens et al., 2018; Burow et al., 2019). In order to better understand the relationships between AMU and AMR, both qualitative and quantitative measurements of AMU are needed (Collineau et al., 2017; Stebler et al., 2019).

Attempts to harmonize the collection of both AMU (Werner et al., 2018) and AMR data (WHO, 2017) have been made. Many national AMU monitoring programs, however, do not meet the criteria of data collection for scientific purposes. In Finland, the national web-based health and welfare register Sikava provides relatively detailed information about AMU in Finnish pig herds. The register is relatively comprehensive, as altogether 90% of Finnish swine herds have joined Sikava, representing 97% of all pork production in Finland (Animal Health ETT, 2021. Individual Sikava herds need to make a healthcare agreement with their herd health veterinarian, who is responsible for planning and providing advice on medicine use in the herd and also for documenting his/her health and disease-related findings after regular herd health visits. One of the many prerequisites for the Sikava herds is to save their medicine use in the electronic Sikava system. Consequently, it allows the calculation of herd-level AMU separately for different pig age groups.

Different ways to quantify AMU result in different outcomes (Taverne et al., 2015; O’Neill et al., 2020). To standardize the calculations, various technical units have been developed (Collineau et al., 2017). The population at risk of being treated with antimicrobials is used to evaluate the exposure of the population to antimicrobials and is indicative of general AMR selection pressure (Collineau et al., 2017). In that context, the population under investigation can be expressed either as biomass based on the live weight of animals (in kilograms) or as the number of individuals at risk (Collineau et al., 2017). Indicator organisms can be utilized to assess the impact of herd-level AMU on the intestinal microbiota of animals (Aarestrup et al., 1998; JIACRA, 2015). *Escherichia coli* bacterium is a widely used indicator organism due to its commensal nature and abundance in the intestines (Aarestrup et al., 1998; EFSA, 2012). Acquired resistance is common in *E. coli* (Aarestrup et al., 1998), and as genes encoding AMR traits can transfer between commensal and pathogenic *Enterobacteriaceae* (Blake et al., 2003); thus, the resistance pattern in *E. coli* is thought to represent most resistance traits found in Gram-negative bacteria in animals (EFSA, 2012).

In the present study, we aimed to describe qualitatively and quantitatively AMU in selected Finnish piglet-producing and finishing herds that formed nine birth-to-slaughter production lines. We described AMU separately for different pig age groups and assessed the proportion of resistant indicator *E. coli* and phenotypic AMR pattern in these herds. Finally, we investigated the associations between AMU and AMR at herd level and covering the birth-to-slaughter production lines. We hypothesized that overall AMU contributes to herd-level AMR and within each production line, and AMU in piglet-producing herds influences the resistance status of the finishing herds. We also hypothesized that use of different antimicrobial groups has an effect on the phenotypic resistance pattern observed in these herds. Furthermore, we predicted that the proportion of resistant indicator *E. coli* is higher in large herds than in smaller ones due to presumptive higher AMU.

## Materials and Methods

### Selection of Study Herds and Herd Characteristics

The study included a convenience sample of 25 pig herds that were recruited by the Finnish slaughterhouse company A-Tuottajat Plc with the following criteria: location in South and South-Western Finland, integrated production from birth to slaughter, and participation in the Sikava. The herds formed nine production lines of which each included one piglet producer and 1–3 finishing herds. Ten piglet-producing herds (P1-P10) sold their piglets at about 30kg body weight to 15 finishing herds (F1-F15), where the pigs were reared to about 110kg body weight. One of the piglet-producing herds (P10) bought weaned piglets weighing on average 7kg and reared them to about 30kg body weight.

The monthly number of pigs present on each herd for the year 2018 was obtained from the Finnish Swine Registry system authorized by the Finnish Food Authority. Farmers are obliged to report their animal numbers, including suckling piglets, weaned piglets, sows, boars, young breeder pigs (gilts), and finishing pigs, to the system. In 2018, the median number of sows in P1-P9 was 896 (range 248–3,422), the total number of weaned piglets raised in P10 was 83,911, and the median number of finishing pigs farmed in F1-F15 was 13,320 (range 3,431–47,255).

### Collection and Calculations of AMU

Treatment records of each herd for the year 2018 were collected from the Sikava register, where the herd owners saved their medicine use in electronic form. Data on antimicrobial treatments were extracted among all treatment records, and the AMU data included age group-specific information on the number of pigs treated, antimicrobial products used, doses, and administration routes (injectable or oral). Age groups were suckling piglets, weaned piglets, finishing pigs, sows, boars, and young breeding pigs. Antimicrobial agents used (penicillin, amoxicillin, sulfadiazine/−doxine in combination with trimethoprim, enrofloxacin, marbofloxacin, danofloxacin, oxytetracycline, chlortetracycline, tylosin, long-acting tulathromycin, lincomycin, and tiamulin) were grouped as follows: penicillin, beta-lactams other than penicillin, sulfa-trimethoprim, fluoroquinolones, tetracyclines, macrolides, lincosamides, and pleuromutilins.

The total biomass of suckling and weaned piglets, finishing pigs, and young breeding pigs was obtained by summarizing the monthly numbers of each group multiplied by the standard weight of the animals. For sows and boars, the average monthly number of animals over 12months was calculated. The standard weights used for calculations were as follows: 4kg for suckling piglets, 12kg for weaned piglets, 50kg for finishing pigs, and 220kg for sows (EMA, 2013). The corresponding defined days at risk were set at 28, 42, 130, and 365days, those being the time periods when a pig could receive an antimicrobial treatment in each age group. For total AMU calculations, treatments of all age groups reported to the Finnish Swine Registry were included with two exceptions: In five piglet-producing herds, treatments concerning 1739 finishing pigs (0.9% of all treatments) were deleted from the data because no finishing pigs were reported to exist on these farms, and in one piglet-producing herd, treatment records were available only from the farrowing unit, which resulted in a slight underestimation of AMU for the sows in this herd. The population correction unit was adjusted to total AMU quantification at herd level and at age group level according to the European Medicines Agency (EMA) report (EMA, 2011). PCU is a technical unit of measurement obtained from the number of treated animals within the animal category multiplied by the standard weight of the animals corresponding to the age at the time of treatment (EMA, 2011). The use of different antimicrobial groups per herd was quantified as milligrams based on the concentration given on the product label and the administered dose. At individual pig level, treatment incidences (TIs) were calculated for each age group and separately for antimicrobial groups used for each age group, according to Timmerman et al. (2006) (Formula 1). Defined daily dose (DDD) values needed for the TI calculations were obtained from the publication of Postma et al. (2015), as it includes the DDD value also for long-acting macrolides.


$$\begin{array}{l} \mathrm{TI}\left(\frac{\mathrm{amount}\;\mathrm{of}\;\mathrm{antimicrobial}\;\mathrm{active}\;\mathrm{substance}\left(\mathrm{mg}\right)}{\begin{array}{l} \mathrm{DDD}\left(\frac{\mathrm{mg}}{\mathrm{kg}}\right)\times \;\mathrm{days}\;\mathrm{at}\;\mathrm{risk}\left(d\right)\\ \;\;\;\;\;\;\;\;\;\;\;\;\;\;\;\;\;\;\;\;\times \mathrm{number}\;\mathrm{of}\;\mathrm{pigs}\;\mathrm{treated}\\ \;\;\;\;\;\;\;\;\;\;\;\;\;\;\;\;\;\;\;\;\;\;\;\;\;\times \mathrm{standard}\;\mathrm{weight}\;\left(\mathrm{kg}\right) \end{array}}\right)\\ \;\;\;\;\;\times \mathrm{1000}\;\mathrm{pigs}\;\mathrm{at}\;\mathrm{risk} \end{array}$$

Formula 1 Treatment incidence (TI) calculation according to Timmerman et al. (2006).

### Herd Visits and Fecal Sampling of Study Pigs

The study herds were visited once between March and October 2018. For feces collection, one room housing the oldest weaned piglets in P1-P10 and one room housing the oldest finishing pigs in F1-F15 was selected. Ten evenly distributed pens were sampled (one pooled sample per pen) that in piglet-producing herds contained on average 18 weaned piglets and in finishing herds 6–15 pigs per pen. Sick pens were excluded. Altogether, 250 pooled samples were taken, and for one sample, three fresh feces piles were swabbed with one sterile cotton swab that was inserted into a culture medium tube (M40 Transystem Amies Agar Gel, Copan Diagnostics, Brescia, Italy). Samples were stored inside a cool box and transported to the laboratory, where the sample analysis started within a median of two (range 1–6) days.

### Laboratory Analyses

Samples were enriched in buffered peptone water (BPW) (Oxoid, Basingstoke, Hampshire, UK) and incubated at 37°C for 16–20h. A loopful (10μl) of enriched sample was cultured on selective media (CHROMagar^™^ Orientation, Paris, France) and incubated at 37°C for 20–24h. A typical *E. coli* colony was picked from each plate (250 plates from altogether 25 herds; 10 isolates picked per herd) and streaked on trypticase soy agar plates (Sigma Aldrich, St. Louis, MO, United States) and incubated at 37°C for 20–24h. The colonies were confirmed as *E. coli* by Gram-staining, oxidase test, and API 20 E biochemical test assay (Biomérieux, Marcy-L Étoile, France). The isolates (*n*=250) were stored at −80°C until susceptibility testing.

Broth microdilution (Sensititre^™^ panels, TREK diagnostic systems, Cleveland, OH, United States) was used for the antimicrobial susceptibility testing of indicator *E. coli* according to the manufacturer’s instructions. Phenotypic susceptibility was determined by using minimal inhibitory concentration values for the following 14 antimicrobials: ampicillin, azithromycin, cefotaxime, ceftazidime, chloramphenicol, ciprofloxacin, colistin, gentamicin, meropenem, nalidixic acid, sulfamethoxazole, tetracycline, tigecycline, and trimethoprim. For the quality control, *E. coli* ATCC 25922 was used as a reference strain. The susceptibility results were categorized as either wild type (with reference to susceptible) or non-wild type (with reference to resistant) based on epidemiological cutoff values (ECOFFs) defined by the European Committee on Antimicrobial Susceptibility Testing (EUCAST, 2021). Because no ECOFF value for azithromycin was available, a value suggested by the European Food Safety Authority (EFSA) was used (EFSA, 2020b). Isolates displaying resistance to at least three antimicrobial groups were considered multi-resistant (MR) according to Schwarz et al. (2010). Sampling procedure and analyses are illustrated in Supplementary Figure S1.

### Statistical Analysis

STATA (version 16.1, StataCorp, College Station, TX, United States) was used for statistical processing of the data, and herd was used as the experimental unit in all statistical analyses. Data normality was visualized with histograms. Quantification of AMU was done for the year 2018, and antimicrobial susceptibility testing for each herd was conducted once within that year. We expected, however, that the AMU of the study herds is steady throughout the year, thus justifying the comparison between longitudinal AMU and cross-sectional AMR parameters.

#### Variable Definitions and Data Manipulation for Statistical Analyses

The number of different antimicrobial active substances used in a herd and the number of different AMR phenotypes were categorized into three classes: 1–2, 3–4, and 5–6 and 1, 2, and ≥3, respectively. For comparisons including total AMU, it was quantified as mg/PCU. The proportion of resistant isolates in the study herds was calculated by dividing the number of resistant isolates with the total number of studied isolates per herd (*n*=10). Herd AMR was calculated by dividing the number of different antimicrobial agents included in the susceptibility testing panel to which studied isolates were resistant with the total number of antimicrobial agents (*n*=14). The percentage of phenotypes that were the same both in piglet-producing and finishing herds out of all phenotypes detected in piglet-producing herds was calculated to investigate phenotypic resistance at production line level. Three most common phenotypes present in both herd types within a production line (TET, TMX-TMP-TET, and SMX-TMP-TET-AMP, see Figure 3) were included in the analysis to study whether AMU in piglet-producing herd contributed to their presence in piglet-producing and finishing herds.

**Figure 1:**
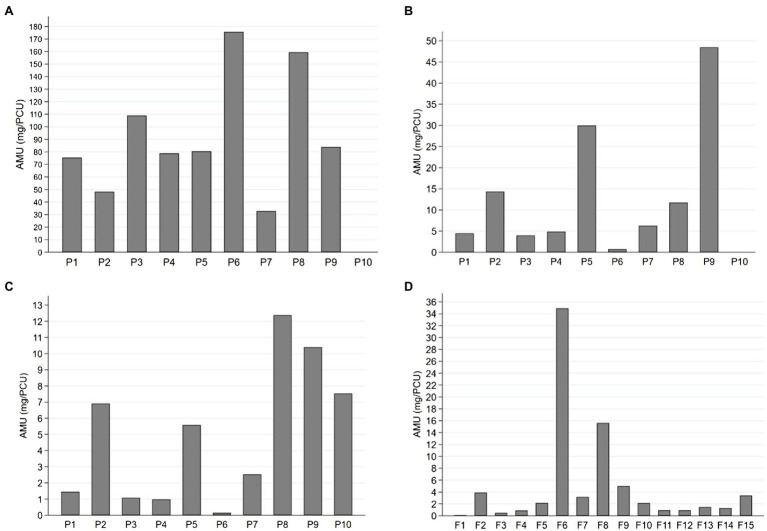
Antimicrobial use (AMU) expressed as milligrams per population correction unit (mg/PCU) for **(A)** sows, **(B)** suckling piglets, **(C)** weaned piglets, and **(D)** finishing pigs in 25 pig herds over 1year. Sows and suckling piglets were treated in 9 piglet-producing herds (P1-P9), weaned piglets in 10 piglet-producing herds (P1-P10), and finishing pigs in 15 separate finishing herds (F1-F15). Note the different scaling of the y-axis for each age group.

**Figure 2:**
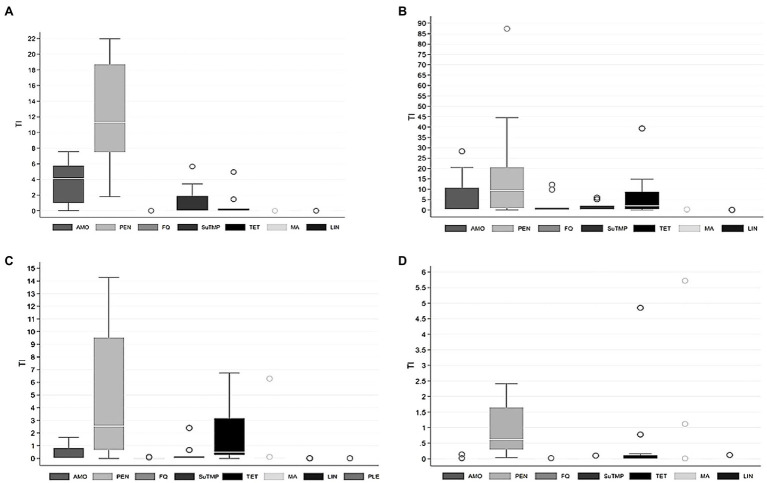
Treatment incidence (TI) by different groups of antimicrobial agents used for **(A)** sows, **(B)** suckling piglets, **(C)** weaned piglets, and **(D)** finishing pigs in 25 pig herds over 1year. Sows and suckling piglets were treated in 9 piglet-producing herds, weaned piglets in 10 piglet-producing herds, and finishing pigs in 15 separate finishing herds. AMO, amoxicillin; PEN, penicillin; FQ, fluoroquinolones; SuTMP, sulfa-trimethoprim; TET, tetracyclines; MA, macrolides; LIN, lincosamides; PLE, pleuromutilins. Note the different scaling of the y-axis for each age group.

**Figure 3:**
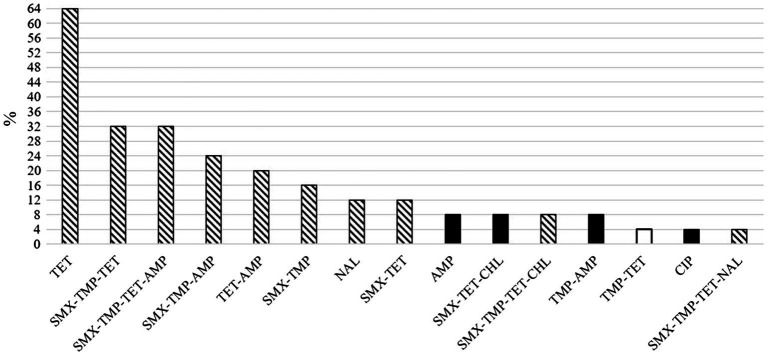
Proportions of herds of 25 total in which the different resistance phenotypes of indicator *Escherichia coli* were detected in pig feces. The white column represents the unique phenotype found in one piglet-producing herd and the black columns four unique phenotypes found in finishing herds. The columns with pattern fill represent ten phenotypes detected in both herd types. AMP, ampicillin; CHL, chloramphenicol; CIP, ciprofloxacin; NAL, nalidixic acid; SMX, sulfamethoxazole; TET, tetracycline, TMP, trimethoprim.

#### Associations

For pairwise comparisons of multiple AMU and AMR variables, Spearman correlations were used with Bonferroni correction.

The difference in the number of different antimicrobial agents used in piglet-producing and finishing herds was tested with Mann–Whitney U test. The association between herd size and AMU (mg/PCU) of the herd was tested with Spearman correlation. Similarly, we tested whether the average number of sows and the total number of suckling and weaned piglets were associated with AMU (mg/PCU) of the respective age groups.

For the outcomes “proportion of resistant isolates in the study herds” and “herd AMR,” linear regression models were built for piglet-producing herds and mixed effect regression models for finishing herds. Models for piglet-producing herds contained total AMU per herd and herd size as fixed effects. For finishing herds, production line was included as a random intercept in both models. In piglet-producing herds, the associations between AMU (mg/PCU) for separate age groups, the proportion of resistant isolates, and herd AMR were investigated with Spearman correlations. For the same outcomes, we examined whether the use of separate antimicrobial groups as milligrams and TIs of separate age groups was associated with AMR by using Spearman correlations.

To investigate whether total AMU for both herd types and separately for suckling piglets, weaned piglets, and sows (mg/PCU) were associated with the number of different AMR phenotypes, Spearman correlation was used. Additionally, total use of separate antimicrobial groups as milligrams and TIs separately for all age groups was compared with the number of different resistance phenotypes by using Spearman correlation. The association between the number of different antimicrobial agents used in herds and the number of different resistance phenotypes by using categorical variables was examined with chi-square test. For the binary outcome “occurrence of phenotype X (*n*=15) in a herd,” Spearman correlation was used. The following explanatory variables were included in comparisons: total AMU for both herd types and total use of separate antimicrobial groups as milligrams, use by age group (mg/PCU, TIs), and TIs of different age groups separated by antimicrobial group. Total AMU and number of different antimicrobial agents used in piglet-producing herds were compared with herd AMR and with the presence of different resistance phenotypes (*n*=14) in finishing herds by using Spearman correlation.

Within production lines, the relationships between total AMU and number of different antimicrobial agents in piglet-producing herds and the occurrence of the same phenotypes in finishing herds were evaluated with Spearman correlation. Spearman correlation with the same explanatory variables was used to examine the presence of the most common AMR phenotypes in piglet-producing and finishing herds. Finally, Kruskal-Wallis test was applied to determine whether the proportion of phenotypes that were the same in both piglet-producing and finishing herds of all phenotypes detected in piglet-producing herds differed between production lines.

## Results

### AMU

Altogether 190,212 pigs had been treated with antimicrobials in 1year: 96.8% parenterally and 3.2% orally *via* feed. Suckling piglets were treated only parenterally. Significantly more different antimicrobial agents were used in piglet-producing herds (5, 3–6; median, range) compared to finishing herds (1, 1–4) (Mann–Whitney U test, *p*<0.001). Table 1 summarizes AMU quantified as mg/PCU and as TIs for suckling piglets, weaned piglets, sows, and finishing pigs. Herd size was not associated with total AMU in either herd type. Additionally, no relationship was found between the average number of sows or the total number of suckling or weaned piglets and AMU (mg/PCU) of the respective age groups.

**Table 1:** Descriptive information of antimicrobial use (AMU) groups quantified as milligrams per population correction unit (mg/PCU) and as treatment incidences (TIs) in 25 Finnish pig herds by age group.

Age group	AMU, mg/PCU			TI			N of treated pigs
	Mean (SD)	Median	Min-Max	Mean (SD)	Median	Min-Max	
Suckling piglets (*n*=9 herds)	13.9 (15.6)	6.3	0.7–48.5	38.8 (40.8)	22.5	1.4–118.5	88,172
Weaned piglets (*n*=10 herds)	4.9 (4.3)	4.1	0.2–12.4	8.2 (7.3)	6.3	0.2–21.7	77,022
Sows (*n*=9 herds)	93.7 (47.3)	80.4	32.8–175.7	17.3 (8.2)	15.1	6.2–31.4	15,964
Finishing pigs (*n*=15 herds)	5.1 (9.1)	2.1	0.1–34.9	1.8 (2.2)	1.0	0.1–8.0	6,297

Considerable variation existed in AMU (mg/PCU) between herds and age groups (Figure 1). The use of different antimicrobial groups as TIs by age group is presented in Figure 2, which indicates highly variable pattern of use, although penicillin was consistently the most commonly administered antimicrobial for all age groups.

### Susceptibility Patterns of Indicator *E. coli*

Altogether 59.6% of 250 indicator *E. coli* isolates studied were fully susceptible. Of 101 resistant *E. coli* isolates, 53.5% were R and 46.5% MR. Overall, the average proportion of isolates resistant to at least one antimicrobial agent was 40.4% (SD 15.1%), being 43.0% (SD 17.7%) in piglet-producing herds and 38.7% (SD 13.6%) in finishing herds. Resistance was most common against tetracycline (29.6%), sulfamethoxazole (22.0%), trimethoprim (21.2%), and ampicillin (15.2%) (Supplementary Tables 1 and 2). A few of the isolates was resistant to ciprofloxacin, nalidixic acid, or chloramphenicol, whereas none of the isolates showed resistance to azithromycin, cefotaxime, ceftazidime, colistin, gentamicin, meropenem, or tigecycline.

**Figure 4:**
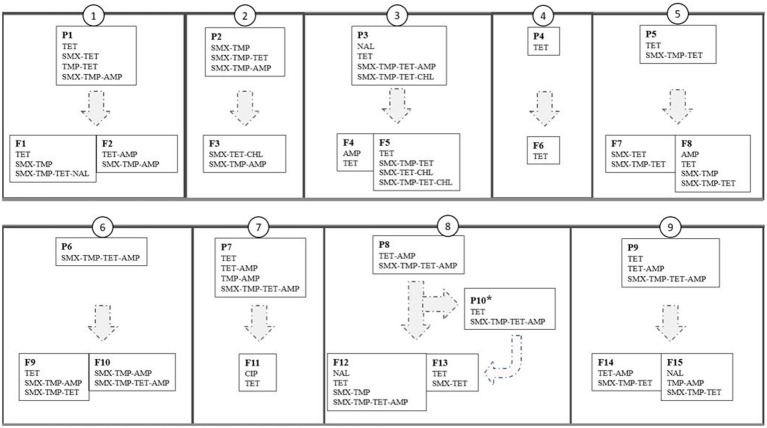
Resistance phenotypes of indicator *Escherichia coli* isolated from pig feces in 25 herds (small boxes with narrow outline) that constitute nine production lines (large boxes with thick outline numbered as 1–9). P1-P9=piglet-producing herds, P10*=weaning unit, F1-F15=finishing herds. AMP, ampicillin; CHL, chloramphenicol; CIP, ciprofloxacin; NAL, nalidixic acid; SMX, sulfamethoxazole; TET, tetracycline; TMP, trimethoprim.

Overall, the resistance patterns of the studied isolates comprised 15 different AMR phenotypes (11 and 14 in piglet-producing and finishing herds, respectively, Figure 3) at herd level. One to four different AMR phenotypes were found, and at least one MR isolate was detected in 20 (80.0%) of the 25 study herds. The MR isolates were resistant to either three or four different antimicrobial agents (Figure 4).

In an average of 60.6% (SD 28.8%) of the production lines, the resistance phenotypes of indicator *E. coli* were the same in piglet-producing and finishing herds of the same line. The difference between lines was statistically significant (Kruskal-Wallis, *p*<0.01).

### AMU and Resistance at Herd Level

Total AMU, AMU at age group level (mg/PCU, TIs), or number of different antimicrobials used were not associated with AMR outcomes, including proportion of resistant isolates in the study herds, herd AMR, number of different resistance phenotypes, or the presence of single AMR phenotypes. In finishing herds, total AMU was negatively associated with herd AMR (mixed effects ML regression, coefficient −0.006, *p*<0.01).

At antimicrobial group level, when AMU was quantified as milligrams or TIs per age group, significant associations between AMU and the presence of single AMR phenotypes were found. The presence of seven AMR phenotypes was influenced by the use of different antimicrobial groups, except for pleuromutilins. The significant associations are shown in detail in Table 2.

**Table 2:** Associations between the use of different antimicrobial groups to treat pigs and resistance phenotypes of indicator *Escherichia coli* found in 25 study herds.

Antimicrobial group	Level of quantification, unit	Resistance phenotype	Spearman correlation coefficient and value of p^**^
Amoxicillin^*^	Finishing pigs, TI	SMX-TMP-TET-NAL	*r*=0.63, *p*<0.05
Penicillin	Total use, mg	SMX-TMP-TET-AMP	*r*=0.45, *p*<0.05
	Weaned piglets, TI	TET-AMP	*r*=0.65, *p*<0.05
Fluoroquinolones	Finishing pigs, TI	SMX-TMP	*r*=0.53, *p*<0.05
Sulfa-trimethoprim	Total use, mg	SMX-TMP-TET-AMP	*r*=0.50, *p*<0.05
	Total use, mg	TET-AMP	*r*=0.42, *p*<0.05
	Suckling piglets, TI	TET	*r*=− 0.76, *p*<0.05
	Weaned piglets, TI	TET	*r*=− 0.90, *p*<0.01
	Sows, TI	SMX-TMP-TET-AMP	*r*=0.76, *p*<0.05
	Finishing pigs, TI	SMX-TET	*r*=0.68, *p*<0.05
Tetracyclines	Finishing pigs, TI	AMP	*r*=0.52, *p*<0.05
Macrolides	Total use, mg	AMP	*r*=0.52, *p*=0.01
Lincosamides	Total use, mg	TET-AMP	*r*=0.56, *p*<0.01
	Weaned piglets, TI	TET-AMP	*r*=0.76, *p*<0.05
	Finishing pigs, TI	SMX-TET	*r*=0.68, *p*<0.05

*Antimicrobial use is quantified either as total use in herds in milligrams (mg) or as treatment incidences (TIs) per age group. SMX, sulfamethoxazole; TMP, trimethoprim; TET, tetracycline; NAL, nalidixic acid; AMP, ampicillin*.

* *Amoxicillin belongs to the group beta-lactams other than penicillin*.

** *value of p are Bonferroni – corrected.*

### AMU and Resistance in Production Lines

Total AMU or number of different antimicrobial agents used in the piglet-producing herds (1–6 per herd) was not associated with the herd AMR in finishing herds or with the presence of any particular resistance phenotype. There was a negative relationship between the number of different antimicrobial agents used in piglet-producing herds and the occurrence of the same phenotypes in the finishing herds (Spearman correlation, *r*=−0.79, *p*<0.001) and the presence of the SMX-TMP-TET-AMP phenotype (Spearman correlation, *r*=−0.94, *p*<0.001) in finishing herds. Moreover, total AMU in piglet-producing herds was negatively associated with the presence of TET phenotype in both herd types (Spearman correlation, *r*=−0.70, *p*<0.05).

## Discussion

Our results indicate that the vast majority of all antimicrobial treatments are given to individual pigs parenterally in the examined herds, whereas oral antimicrobials constituted a minor proportion (3.2%) of all pigs treated. Even though AMU of the individual study herds was rather variable, narrow-spectrum beta-lactams, especially penicillin, was used predominantly in each herd. This implies that both the administration route and the selection of antimicrobials for treatment followed national (Finnish Food Authority, 2018) and European (EMA, 2019) prudent AMU guidelines. More than half of the *E. coli* isolates were fully susceptible, and the resistant ones showed a fairly uniform resistance pattern: resistance to tetracycline, sulfamethoxazole, trimethoprim, and ampicillin common and representative of the major antimicrobial groups used in the herds. Such a good phenotypic susceptibility pattern supports further investigations regarding narrow-spectrum antimicrobials and antimicrobial administration route. However, the associations between AMU and AMR were scarce and mainly present at antimicrobial group level. Different age groups ranked divergently in their AMU depending on the quantification method, and we were unable to show whether AMU in piglet-producing herds contributes to AMR in finishing herds of the same pork production line.

Suckling piglets was the only age group that was treated only parenterally yet parenteral treatment predominated through all age groups. Most of the oral treatments were administered to weaned and finishing pigs and *via* feed, whereas no antimicrobials were given *via* drinking water. Contrary to our results, prophylactic oral group treatments are reported to be common, especially during the post-weaning period in many European countries (Callens et al., 2012; Moreno, 2012, 2014; Sjölund et al., 2016; Hémonic et al., 2018; Lekagul et al., 2019; Sarrazin et al., 2019; O’Neill et al., 2020) and in North America (Akwar et al., 2008). Therapeutic instead of prophylactic use is recommended in the Finnish national guidelines (Finnish Food Authority, 2018) and in Europe (EMA, 2019), and our results are in line with these recommendations.

Penicillin is a common antimicrobial agent used for pigs (Akwar et al., 2008; Jensen et al., 2012; Hémonic et al., 2018; Lekagul et al., 2019; O’Neill et al., 2020; Yun et al., 2021) and recommended as the first-choice antimicrobial agent for the treatment of relevant infectious pig diseases in Finland (Finnish Food Authority, 2018). Similar to our findings, a recent Finnish study of Yun et al. (2021) showed that penicillin was used in every herd and for all age groups in most piglet-producing herds and also Swedish herds relied on penicillin and favored injectable antimicrobials (Sjölund et al., 2016). Other European countries have reported more frequent use of aminopenicillins (including amoxicillin), especially in weaned piglets (Sjölund et al., 2016), whereas amoxicillin was used mainly for suckling piglets and sows in our study herds. Both tetracyclines (Jensen et al., 2012; Moreno, 2012; Lekagul et al., 2019; Yang et al., 2019; O’Neill et al., 2020) and sulfa-trimethoprim (Jensen et al., 2012; O’Neill et al., 2020; Yun et al., 2021) are also commonly used in pigs. In Finland, use of tetracyclines has followed a constant rate and is among the lowest in Europe (Garcia-Migura et al., 2014; ESVAC, 2020). In the present study, the use of tetracyclines was rather low and it was given mostly to suckling and weaned piglets. Tetracyclines are not recommended as a primary treatment option for any relevant pig disease in Finland (Finnish Food Authority, 2018), mostly because of its broad-spectrum nature and the probability of many swine pathogens being resistant to it. Sulfa-trimethoprim, however, is listed as a treatment option for many conditions in sows (Finnish Food Authority, 2018). In the present study, the use of sulfa-trimethoprim for sows was in accordance with recommendations.

Fluoroquinolones were used in very low amounts in the study herds, and 3rd-generation cephalosporines were not used at all. These results are not surprising because their use is restricted by Finnish national legislation and justified only if no other effective treatment exists (Ministry of Agriculture and Forestry, 2014). The same trend has been reported from other Nordic countries (Jensen et al., 2012; Sjölund et al., 2016; ESVAC, 2020). By contrast, injectable fluoroquinolones had been used in 83.6% of pig herds according to an Irish investigation (O’Neill et al., 2020). The use of critically important antimicrobials being reserved for humans (WHO, 2019b), not animals (EMA, 2019), is more common in some other European countries (De Briyne et al., 2014; Sjölund et al., 2016). Nevertheless, especially in the participating piglet-producing herds, we identified that many different antimicrobial substances were used, which corresponds to the reports from other countries (Akwar et al., 2008; Makita et al., 2016; Hémonic et al., 2018; O’Neill et al., 2020). As several age groups and pathogens are present in piglet-producing herds, the need to use different medicines is understandable. However, it should be asked whether different agents are really needed and whether treatments in herds could be planned with fewer active substances without compromising the treatment effect.

Likewise in other studies (Chauvin et al., 2008; Jensen et al., 2012; Taverne et al., 2015; Collineau et al., 2017; Sarrazin et al., 2019), we got different results depending on the AMU quantification method. As mg/PCU, sows were the most medicated age group, followed by suckling and weaned piglets and finishing pigs. Conversely, more suckling piglets were treated than sows as TIs, and the latter age groups remained in the same order. In terms of the number of treatment records, suckling piglets were treated most with antimicrobials, followed by weaned piglets, sows, and finishing pigs. The last AMU pattern corresponds with other studies, as higher use in younger pigs than in adults has been widely reported (Jensen et al., 2012; Sjölund et al., 2016; Raasch et al., 2018; Lekagul et al., 2019; Sarrazin et al., 2019; Yun et al., 2021).

It is challenging to find studies in which AMU has been quantified uniformly. In many pig studies, TI has been applied to assess AMU (Timmerman et al., 2006; Dewulf et al., 2007; Raasch et al., 2018; O’Neill et al., 2020; Yun et al., 2021), but comparable standard weights have been used primarily for sows only. The present investigation revealed that the TI of sows was surprisingly high (median TI 15.1). In other studies, sows have usually been the among the least medicated age groups (van Rennings et al., 2015; Sjölund et al., 2016; Raasch et al., 2018; O’Neill et al., 2020). For example, the median TI for breeders, including sows, gilts, and boars, in Belgian, German, French, and Swedish herds was 6.1, 0.7, 21.1, and 8.4, respectively (Sjölund et al., 2016). Remarkably, sows were also the second most medicated age group in Swedish herds (Sjölund et al., 2016). One could speculate here whether sows are treated at a lower threshold in Nordic countries or whether the standard weight for sows has been set too low. However, the variation in AMU within countries is considerable (Raasch et al., 2018; O’Neill et al., 2020; Yun et al., 2021), suggesting that also other than calculation-related factors, such as herd type (van der Fels-Klerx et al., 2011; Moreno, 2012), could influence the results. Many studies have, for instance, investigated farrow-to-finish herds (Sjölund et al., 2016; Raasch et al., 2018; O’Neill et al., 2020).

Positively, more than half of the studied isolates were susceptible to all antimicrobials tested. A similar trend was reported in Finnish herds recently (Yun et al., 2021) and is in line with the results from the national AMR monitoring (Finnish Food Authority, 2020; EFSA, 2020a). As in Nordic neighbors, the resistance occurrence among porcine indicator *E. coli* in Finland is low relative to other European countries (EFSA, 2020a). The proportion of MR isolates of all resistant *E. coli* was 47% in this study, and these were distributed across both herd types. Nationally, the proportion of MR indicator *E. coli* of all resistant isolates among slaughtered pigs was 36% in 2019 (Finnish Food Authority, 2020). Younger pigs have been reported to carry more resistant bacteria (Langlois et al., 1988; Dewulf et al., 2007; Akwar et al., 2008; Yun et al., 2021), which may explain the observed difference because we took samples from pigs at post-weaning and finishing stages. The MR isolates found, however, were resistant against a maximum of four different antimicrobials. For comparison, MR indicator *E. coli* isolates elsewhere in Europe have been reported to show resistance against 3–8 antimicrobials (EFSA, 2020a).

Overall, the antimicrobial susceptibility pattern resembled the AMU in our study herds. This finding is in line with the literature, as the observed resistance at country level seems to follow the consumption of different antimicrobial groups (Chantziaras et al., 2014; JIACRA, 2015). The greatest proportion of isolates was resistant to tetracycline, followed by sulfamethoxazole, trimethoprim, and ampicillin. Resistance to these antimicrobials has been common in Finland (Finnish Food Authority, 2020; Yun et al., 2021), in Europe (EFSA, 2020a; Mencía-Ares et al., 2021) and elsewhere (Hart et al., 2004; Agga et al., 2014; Yassin et al., 2017). The MR phenotypes detected in our study herds included resistance usually to sulfamethoxazole, trimethoprim, and tetracycline. A similar pattern has been reported previously in Finland (Finnish Food Authority, 2020; Yun et al., 2021) and Europe (EFSA, 2020a). Dewulf et al. (2007) observed that resistance against tetracyclines was often accompanied by resistance against ampicillin and sulfa-trimethoprim. Here, ampicillin resistance was also a relatively common finding.

Fluoroquinolone resistance was rare; a single ciprofloxacin-resistant isolate was found in one finishing herd, constituting 1% of all resistant isolates. According to the latest national report, the proportion of ciprofloxacin-resistant indicator *E. coli* of all studied isolates was 1.7% in slaughtered pigs (Finnish Food Authority, 2020) and followed the constant low rates reported in other Nordic countries (Chantziaras et al., 2014; Garcia-Migura et al., 2014; EFSA, 2020a). In Europe, ciprofloxacin resistance among indicator *E. coli* is variable between countries (EFSA, 2020a). Further, all isolates were susceptible to azithromycin, colistin, gentamicin, and tigecycline, and no resistance against 3rd-generation cephalosporins or meropenem was detected. Resistance against 3rd-generation cephalosporins has not been common in Finland (Finnish Food Authority, 2020; EFSA, 2020a), similar to other Nordic countries (Chantziaras et al., 2014; Garcia-Migura et al., 2014; EFSA, 2020a), which may be linked to prudent AMU in these countries.

Although the results presented above suggest a linkage between AMU and AMR, such relationships are difficult to verify. Similar to our findings, Yun et al. (2021) reported recently that higher AMU in herds was not reflected in a higher proportion of resistant isolates. By contrast, Akwar et al. (2008) showed that AMU in both weaned and finishing pigs was associated with the proportion of resistance to various antimicrobial agents. Perhaps due to the small sample size of this study and the low number of isolates per herd, we were unable to show a positive association between AMU and AMR. We found that total AMU (mg/PCU) was negatively associated with herd AMR only in finishing herds; however, the effect was minor; an increase in AMU by 1mg/kg lowered the resistance of isolates by 0.006units. Possibly, treatments with a few different antimicrobial substances (mostly penicillin) have favored certain strains becoming more prevalent than others over time. Some evidence exists that different antimicrobials influence the development of AMR at differing intensity (Harada et al., 2008; Makita et al., 2016; Birkegård et al., 2017). In addition, we sampled a selected group of pigs per herd, which had presumably experienced the longest possible antimicrobial selection pressure, but might still not be representative of the susceptibility pattern in these herds.

Our investigation revealed significant associations between AMU and presence of certain AMR phenotypes when AMU was quantified at antimicrobial group level as milligrams or as TIs for different age groups (Table 2 in the Results section). Altogether seven AMR phenotypes were significantly associated with AMU. Of these, phenotype SMX-TMP-TET-AMP, which was among the most prevalent phenotypes, showed the clearest association with AMU. This phenotype is very common among indicator *E. coli* isolated from pigs also in other European countries (EFSA, 2020a). Similar to Makita et al. (2016), we found that the AMU was associated with resistance not only to antimicrobials belonging to the same antimicrobial class but also to antimicrobials belonging to other antimicrobial classes. Resistance genes occurring in same mobile genetic elements and mobile gene cassettes (Gillings et al., 2008; Deng et al., 2015; Partridge et al., 2018) would explain the coincidence of observed AMR phenotypes and should be addressed more in detail in further investigations. However, not all associations between AMU and AMR phenotypes in this study could be explained, for example, sulfa-trimethoprim use (as TIs) in suckling and weaned piglets was negatively associated with the presence of TET phenotype. In other studies, both corresponding and divergent associations between AMU and AMR, including direct and implicit resistance selection mechanisms, have been reported (Dewulf et al., 2007; Rosengren et al., 2007; Akwar et al., 2008; Harada et al., 2008; Makita et al., 2016). Neither AMU nor AMR has, however, been studied similarly between studies, and consequently, comparison between studies is not possible.

The average proportion of isolates resistant to at least one antimicrobial agent was slightly greater in piglet-producing herds than in finishing herds. We hypothesized that AMU during early production phases would influence AMR until the finishing phase, and depending on the antimicrobial repertoire used in individual herds, different phenotypic AMR patterns could be seen. We observed similar phenotypic resistance profiles in both herd types although the amount of antimicrobials and the number of different antimicrobial agents were lower and the antimicrobial repertoire narrower in finishing herds than in piglet-producing herds. In any case, we did not find any linkage between AMU in piglet-producing herds and herd antimicrobial resistance in the corresponding finishing herds. We also noted that when the number of different antimicrobials used increased in piglet-producing herds, the proportion of the same phenotypes in both herd types diminished and the occurrence of the SMX-TMP-TET-AMP phenotype in finishing herds declined. Bacteria resistant to certain antimicrobial agents have been observed to persist for years after cessation of the use of these agents (Garcia-Migura et al., 2014). However, both the prevailing and the former antimicrobial selection pressure (Mencía-Ares et al., 2021) together with other factors, such as implementing biosecurity measures (Dewulf et al., 2007; Mencía-Ares et al., 2021; Yun et al., 2021), influence AMR development.

Many studies have investigated AMU and/or AMR at one specific production stage (Timmerman et al., 2006; Dewulf et al., 2007; Callens et al., 2015), whereas the present investigation covered the entire birth-to-slaughter line. Along with the age of the animals, the route of antimicrobial administration influences AMR development (Akwar et al., 2008). As an example, in-feed medication has been more consistently associated with AMR than use of injectable antimicrobials (Akwar et al., 2008). Oral administration of antimicrobials allows the antimicrobial to interfere directly with the intestinal bacteria resulting in higher selection pressure. Oral treatment courses concern a larger number of animals, whereas injectable antimicrobials are often administered to a single or a few animals. In addition, underdosing of oral antimicrobials occurs (Timmerman et al., 2006), and as the treatment courses are typically longer, this sub-therapeutic and durable selection pressure favors the development of resistance (Holman and Chénier, 2015).

Contrary to our hypothesis, we did not find a positive relationship between herd size and total AMU (mg/PCU) in either piglet-producing or finishing herds. Similarly, herd size was not associated with the proportion of resistant isolates. Likewise, Moreno (2012) did not observe associations between herd size and AMU in either Spanish farrow-to-finish or finishing herds. Contradictory results have been reported in Finnish farrow-to-finish herds (Yun et al., 2021) and in finishing herds (Stygar et al., 2020), resembling the findings of a German study (Raasch et al., 2018). However, none of the studies used a similar definition of herd size and/or AMU quantification method.

The present study included a convenience sample of herds from selected geographical areas and with integrated production. Although the Sikava register has relatively detailed data on AMU, it may contain some erroneous treatment records. In addition, general practice among farmers is to maintain their medical bookkeeping first on paper, then transferring it to the register in electronic form. To investigate the associations between AMU and AMR, more detailed AMU quantification methods are needed. Currently, there is no harmonized and precise AMU indicator at herd level (Collineau et al., 2017; Lekagul et al., 2019), especially for scientific purposes. Future AMR studies should aim for more widespread investigation, including several herds and age groups and more samples per herd from different locations.

## Conclusion

Our findings indicate that primarily narrow-spectrum beta-lactam antimicrobials were administered parenterally to single pigs. A surprising finding was higher AMU in sows than in other age groups. Almost half of the indicator *E. coli* bacteria studied were fully susceptible, and overall, the resistance pattern followed a trend similar to those reported both nationally and in Europe over several years. Resistance was most common to the antimicrobials generally used in pigs. A novel feature in the present study is that we evaluated the herd-level phenotypic AMR pattern throughout the pork production lines and included a comparison between production lines. Our results did not reveal a positive association between herd-level AMU and AMR. The linkage between AMU and AMR is complicated, and thus, any information shedding light on AMR development is valuable. Taken together, it is important to standardize the unit of AMU measurements and to assess the impact of AMU selection pressure on AMR development in more detail.

## Data Availability Statement

The original contributions presented in the study are included in the article/Supplementary Material, further inquiries can be directed to the corresponding author.

## Ethics Statement

The animal study was reviewed and approved by Southern Finland Regional State Administrative Agency (ESAVI/16950/2018). Written informed consent was obtained from the owners for the participation of their animals in this study.

## Author Contributions

VS, SN, AH, TT, and MH participated in the planning of the study. VS, TT, and MH conducted the herd visits. VS and OH performed the statistical analyses. All authors participated in the writing process and approved the final manuscript.

## Funding

This research was funded by the Finnish Centre for Economic Development, Transport, and the Environment (project number 58451) as part of the Rural Development Program for Mainland Finland 2014–2020 and by the Walter Ehrström Foundation.

## Conflict of Interest

The Finnish slaughterhouse company Atria Finland Plc participated in the study by recruiting the study herds but did not influence the interpretation or reporting of results. One of the authors (TT) is employed by the slaughterhouse company.

The remaining authors declare that the research was conducted in the absence of any commercial or financial relationships that could be construed as a potential conflict of interest.

## Publisher’s Note

All claims expressed in this article are solely those of the authors and do not necessarily represent those of their affiliated organizations, or those of the publisher, the editors and the reviewers. Any product that may be evaluated in this article, or claim that may be made by its manufacturer, is not guaranteed or endorsed by the publisher.
