# Supplementary material for: Antimicrobial Use and Susceptibility of Indicator Escherichia coli in Finnish Integrated Pork Production
Source: Front Microbiol. 2021 Nov 4;12:754894. doi: 10.3389/fmicb.2021.754894 (PMC8600236; doi:10.3389/fmicb.2021.754894)
Supplement: Supplementary file 1 [file Data_Sheet_1.docx]

Supplementary Material

Table 1. Antimicrobial susceptibility results to 14 antimicrobials of 250 indicator *E. coli* isolates (10 isolates per herd) originating from 25 Finnish pig herds. AMP = ampicillin, AZI = azithromycin, CIP = ciprofloxacin, CHL = chloramphenicol, COL = colistin, FOT = cefotaxime, GEN = gentamicin, MERO = meropenem, NAL = nalidixic acid, SMX = sulfamethoxazole, TAZ = ceftazidime, TET = tetracycline, TGC = tigecycline, TMP = trimethoprim.

|  | AMP | AZI^2)^ | CHL | CIP | COL | FOT | GEN | MERO | NAL | SMX | TAZ | TET | TGC | TMP |
| --- | --- | --- | --- | --- | --- | --- | --- | --- | --- | --- | --- | --- | --- | --- |
| ECOFF^1)^ | >8 | >16 | >16 | >0.064 | >2 | >0.25 | >2 | >0.125 | >16 | >64 | >0.5 | >8 | >0.5 | >2 |
| Proportion of resistant isolates (%) | 15.2 | 0 | 1.6 | 0.4 | 0 | 0 | 0 | 0 | 1.6 | 22.0 | 0 | 29.6 | 0 | 21.2 |

^1)^ Epidemiological Cut Off (ECOFF) values for indicator *Escherichia coli* available at 25.1.2019 were used. Available at: European Committee on Antimicrobial Susceptibility Testing. MIC and zone diameter distributions and ECOFFs. https://www.eucast.org/mic_distributions_and_ecoffs/

^2)^ European Food Safety Authority. Annexes to the European Union Summary Report on Antimicrobial Resistance in zoonotic and indicator bacteria from humans, animals and food in 2017/2018 (2020). https://zenodo.org/record/3628719#.YNV21-gzY2w [Accessed April 5, 2020].

Table 2. Proportions of resistant indicator *E. coli* isolates (n = 10 per herd) originating from 25 Finnish pig herds separated by herd, herd type (piglet-producing P1-10 or finishing herd F1-15) and production line (n = 9). AMP = ampicillin, CIP = ciprofloxacin, NAL = nalidixic acid, SMX = sulfamethoxazole, TET = tetracycline, TMP = trimethoprim. Antimicrobials to which any of the isolates were resistant are omitted from the table.

|  | | Antimicrobial class and EFOFF^1)^ -value | | | | | |
| --- | --- | --- | --- | --- | --- | --- | --- |
|  |  | AMP  >8 | CIP  >0.064 | NAL  >16 | SMX  >64 | TET  >8 | TMP  >2 |
| Line number | Herd  abbreviation |  |  |  |  |  |  |
| 1 | P1 | 20 | 0 | 0 | 30 | 40 | 30 |
|  | F1 | 0 | 0 | 10 | 20 | 20 | 20 |
|  | F2 | 30 | 0 | 0 | 10 | 20 | 10 |
| 2 | P2 | 20 | 0 | 0 | 50 | 10 | 50 |
|  | F3 | 20 | 0 | 0 | 30 | 10 | 20 |
| 3 | P3 | 20 | 0 | 10 | 30 | 50 | 30 |
|  | F4 | 40 | 0 | 0 | 0 | 10 | 0 |
|  | F5 | 0 | 0 | 0 | 40 | 60 | 30 |
| 4 | P4 | 0 | 0 | 0 | 0 | 30 | 0 |
|  | F6 | 0 | 0 | 0 | 0 | 20 | 0 |
| 5 | P5 | 0 | 0 | 0 | 20 | 70 | 20 |
|  | F7 | 0 | 0 | 0 | 40 | 40 | 30 |
|  | F8 | 10 | 0 | 0 | 20 | 20 | 20 |
| 6 | P6 | 30 | 0 | 0 | 30 | 30 | 30 |
|  | F9 | 20 | 0 | 0 | 50 | 40 | 50 |
|  | F10 | 40 | 0 | 0 | 40 | 20 | 40 |
| 7 | P7 | 30 | 0 | 0 | 10 | 40 | 20 |
|  | F11 | 0 | 10 | 0 | 0 | 10 | 0 |
| 8 | P8 | 20 | 0 | 0 | 10 | 20 | 10 |
|  | F12 | 10 | 0 | 10 | 20 | 20 | 20 |
|  | F13 | 0 | 0 | 0 | 10 | 20 | 0 |
|  | *P10 | 10 | 0 | 0 | 10 | 20 | 10 |
| 9 | P9 | 30 | 0 | 0 | 20 | 40 | 20 |
|  | F14 | 20 | 0 | 0 | 30 | 50 | 30 |
|  | F15 | 10 | 0 | 10 | 30 | 30 | 40 |

* Weaning unit, considered piglet-producing herd

** All the isolates were susceptible to azithromycin, colistin, gentamicin, and tigecycline and additionally, no resistance against 3^rd^ generation cephalosporins (cefotaxime, ceftazidime) or meropenem was detected.

^1)^ Epidemiological Cut Off (ECOFF) values for indicator Escherichia coli available at 25.1.2019 were used. Available at: European Committee on Antimicrobial Susceptibility Testing. MIC and zone diameter distributions and ECOFFs. https://www.eucast.org/mic_distributions_and_ecoffs/


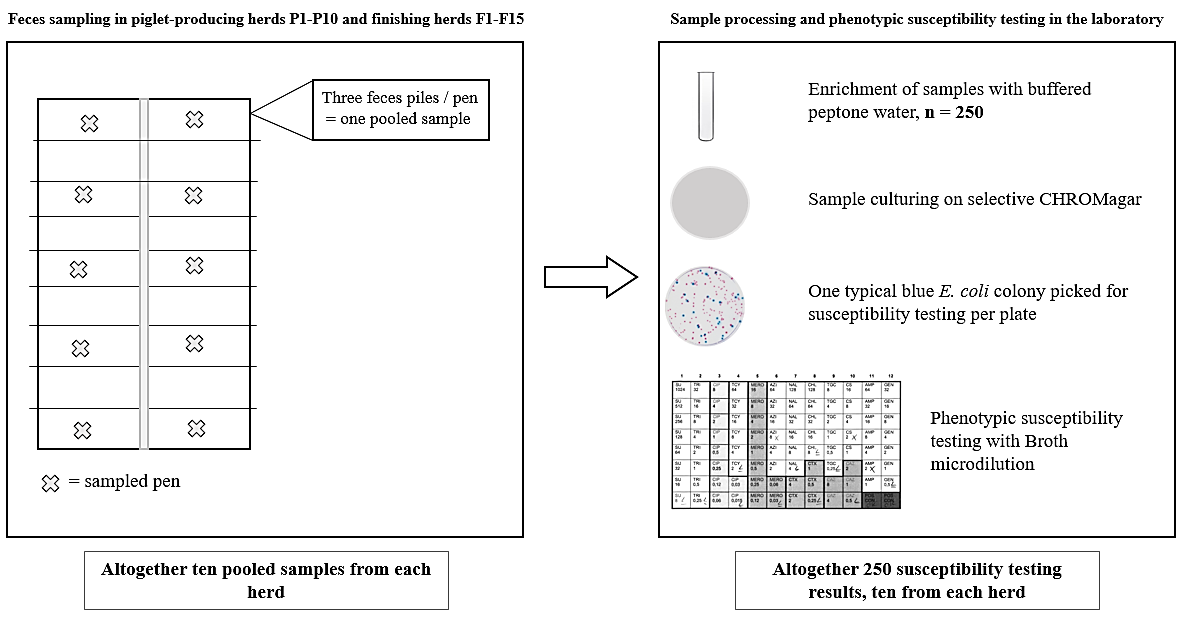


Figure 1. Illustration of feces sampling in 25 Finnish pig herds including ten piglet - producing and fifteen finishing herds and simplified summary of laboratory work conducted for those 250 samples taken.
